# Psychological interventions for parents of children with intellectual disabilities to enhance child behavioral outcomes or parental well-being: A systematic review, content analysis and effects

**DOI:** 10.1177/17446295241302857

**Published:** 2024-11-27

**Authors:** Kati Ranta, Heini Saarimäki, Johanna Gummerus, Jael Virtanen, Satu Peltomäki, Elina Kontu

**Affiliations:** Doctoral Program in Psychology and Logopaedics, Faculty of Social Sciences, 7840 Tampere University, Finland; Faculty of Social Sciences, 7840 Tampere University, Finland; Professor in Marketing and director of CERS, 3856 Hanken School of Economics, Finland; Doctoral Programme in School, Education, Society and Culture (SEDUCE), 3835 University of Helsinki, Finland; Doctoral Programme in School, Education, Society and Culture (SEDUCE), 3835 University of Helsinki, Finland; Faculty of Social Sciences, 7840 Tampere University, Finland; Faculty of Social Sciences, 7840 Tampere University, Finland

**Keywords:** child behavior, children with intellectual disabilities, parent well-being, psychological intervention, systematic review

## Abstract

The aim of this review was to identify the type, content, and effectiveness of psychological parenting interventions for parents of children with intellectual disabilities to enhance child behavior and/or parental well-being. A systematic search yielded 21 studies involving 1825 participants. Studies were evaluated according to intervention content, pre- and post-treatment and follow-up effect sizes, and risk of bias. We categorized the interventions into those targeting ‘Child or interaction’ (child behavior, interaction and learning, understanding disability), and those targeting ‘Parent’ (parental well-being) or both themes. All these interventions had positive effects on parental well-being or child behavior. Parental outcomes were improved by interventions targeting ‘Parental well-being’, as well as ‘Child or interaction’. Child behavior showed improvements in programs focusing on ‘Child or interaction’, and in a mindfulness-based parental well-being program. During follow-up, most effects were sustained or further increased, but some studies showed no improvements over the control group.

## Introduction

Many parents of children with intellectual disabilities are stressed and overwhelmed by challenging behavior of their children. This may lead to counter-productive parenting practices and diminished well-being in the families (Hsiao, 2018; Jacobs et al., 2016). Child’s challenging behavior refers to aggression, destructiveness, self-injury, stereotyped mannerisms, and other behaviors which may be harmful to the individual or problematic to other people (Emerson and Einfeld, 2011). The prevalence of challenging behaviors ranged from 48% to 60% in studies involving children with intellectual disabilities in a recent systematic review (Simó-Pinatella et al. 2019).

Parents of children with intellectual or developmental disabilities show high levels of stress, anxiety, and depression in meta-analyses and population-based studies (Scherer et al., 2019, McConnell and Savage, 2015, Marquis et al., 2020). Mothers of children with intellectual disabilities are two to three times more likely to report clinically significant levels of stress, anxiety, and depression than mothers of typically developing children (McConnell and Savage, 2015). Longitudinal studies have reported bidirectional relationships between child behavior and parental stress (Baker et al., 2021). McConnell and Savage (2015) note that a child’s behavior problems largely explain the high rates of psychological distress and dysfunction among families caring for a child with intellectual disabilities. Problems in children’s behavior and parents’ mental health and a lack of support may even lead to maltreatment. For instance, the American Academy of Pediatrics (Legano et al., 2021) reported that the rate of child abuse and neglect is three times higher in children with disabilities than in children developing typically.

Both parent and child mutually influence their bidirectional relationship, behaviors, and mental health (Paschall and Mastergeorge, 2016). Thus, alleviating parents’ stress and children’s behavior problems is essential for the well-being of families and equal opportunities to participate in social activities. Psychological parenting interventions can be divided into behavioral programs and parent-child relationship programs, and programs combining elements from both (Barlow and Stewart-Brown, 2000). Some of these programs add elements to enhance parental well-being. In addition, there are interventions that directly target parents' psychological well-being (Da Paz and Wallander, 2017). Previous reviews have analyzed interventions for parents of children with autism spectrum disorders, developmental disabilities, or other disabilities (Bourke Taylor et al., 2021; Cachia et al. 2016; Chua and Shorey, 2022; Conrad et al., 2021; Da Paz and Wallander, 2017; Osborn et al, 2021; Petrenko, 2013; Ragni et al., 2022; Ruane and Carr, 2019; Sohmaran and Shorey, 2019). Masulani-Mwale and colleagues (2018) narratively reviewed psychosocial interventions for parents of children with intellectual disabilities. These reviews found mainly positive results for child and parent outcomes. Despite this previous work, to our knowledge, the content and estimated effects of psychological interventions for parents of children with intellectual disabilities have not been systematically reviewed. To better understand the effects of interventions on the well-being of the whole family, including children and parents, we review interventions that aim to enhance either child behavioral outcomes or parental well-being. Therefore, the research questions for this systematic review are:

1. What type of psychological parenting interventions have been used for parents of children with intellectual disabilities to enhance children’s behavioral outcomes and/or parental psychological well-being?
2. Is there common content in these interventions?
3. How effective are these interventions in terms of children’s behavioral and parents’ well-being outcomes?

## Methods

This systematic review is reported according to the Preferred Reporting Items for Systematic Reviews and Meta-Analyses (PRISMA) statement (Page et al, 2021). The Prisma checklist is provided in Supplemental Table 1.

### Eligibility criteria

The population, intervention, comparator, and outcome (PICO) framework was used in this review. Studies were included in the review if:

1. Parents of children with intellectual disabilities were reported as participants in the interventions.
2. The interventions were psychological (such as psychoeducation, behavioral or stress coping).
3. The comparison groups were parents receiving no treatment, treatment as usual or they were on a waiting list.
4. The outcome measures included children’s behavior and/or parental psychological well-being (such as general health, mental health, stress, anxiety, or depression).
5. The study was a randomized controlled trial or quasi-experimental design.
6. The study was published in a peer-reviewed journal in English. And
7. The study had been published since 2010.

Studies were excluded if they:

1. Were pharmacological or non-psychological.
2. Were study protocols. Or
3. Were duplicate publications or data had been included in another more comprehensive study.

### Information sources and search strategy

The systematic search was conducted over the period between March 1 and 12, 2023 in seven databases: Academic Search Ultimate, Education Collection, Medline, Psychology Database, PsycINFO, Scopus, and Web of Science. Peer-reviewed journal articles published since 2010 in English were considered. Additional manual searches of websites were conducted.

The search strategy included search terms: child* OR adolescen* OR youth* OR teen* AND intellect* OR developmental OR learning AND disab* AND parent* OR caregiver* OR carer* OR mother* OR father* OR maternal* OR paternal* OR famil* AND program* OR training* OR educati* OR intervention* AND behavio* OR well-being OR wellbeing OR stress* OR depressi* OR anxi* OR mental* health* AND random* control* trial* OR rct* OR quasi* experiment* OR quasi-experiment*.

The first author screened the titles and abstracts of the search results according to the inclusion and exclusion criteria. Three independent reviewers (KR, SP and JV) screened the full texts of the remaining studies to determine the aptness of their inclusion in the review. Discrepancies were resolved through discussions.

### Data collection and items

The first author collected data from each report included in the review. These data were discussed by the group of authors. A template for intervention description and replication (TIDieR) guided the data extraction (Hoffmann et al., 2014). The data items extracted were:

1. Study characteristics: authors, year, country, design, sample size.
2. Population characteristics: participating parents, child age, child diagnosis.
3. Intervention characteristics: theory or framing; content; mode; sessions and schedule; provider.
4. Comparison group characteristics.
5. Outcomes: outcome measures of parental well-being and child behavior, timepoints of measurements, summary statistics of outcome measures for intervention and control groups at pre-test, post-test and follow-up (mean, standard deviation, sample size).

### Content analysis

A content analysis was conducted to identify common themes in the content of the interventions. The content analysis was an iterative process of refining the coding scheme and analysis while analyzing the data. Coded themes were derived from the text data collecting codes under potential themes and subthemes. The themes were compared to each other and in relation to the entire data set. A theme was defined as a coherent integration of the disparate pieces of data that constitute the findings (Vaismoradi et al., 2013).

### Effect size calculations

Effect sizes were calculated for outcome measures of parental well-being and child behavior. Outcome measures of parental well-being contained measures of both psychological illness and psychological well-being (Ryff and Singer, 1996). Parental outcomes were measured by various scales in the areas of anxiety, depression, stress, maladjustment, general health, mental well-being, quality of life, satisfaction with life, hope, self-compassion, self-efficacy, and sense of competence (Abidin, 1983; Beck et al., 1961; Diener et al., 1985; Dumka et al., 1996; Echeburúa et al., 2000; Emser et al., 2016; Goldberg et al., 1997; Johnston and Mash, 1989; Lovibond and Lovibond, 1995; Neff, 2003; Pedersen et al., 1989; Radloff, 1977; Ravens-Sieberer et al., 2001; Ryff, 1989; Snyder et al., 1991; Stein and Riessman, 1980; Tennant et al., 2007; Tobin et al. 1989; Varni et al., 1999; Zigmond and Snaith, 1983). For a list of scales, see Supplemental Table 2. All measures were parent-reported. All the studies included (N=21) measured parental well-being on at least one scale.

Outcome measures of child behavior included scales of behavior, adaptive behavior, child adjustment, developmental behavior, behavior problems, and strengths and difficulties (Achenbach, 2000; Aman and Singh, 1994; Einfeld and Tonge, 2002; Eyberg and Pincus, 1999; Goodman, 1997; Harrison and Oakland, 2003; Rojahn et al., 2012). For a list of scales, see Supplemental Table 3. All studies used parent-reported measures. One study used additional teacher-reported measures, and another study used additional child-reported measures. Fourteen of the 21 studies measured child behavior outcomes.

Effect size calculations varied between the studies, so we re-calculated all effect sizes to allow effect size comparison. Calculations of pre-post intervention effect sizes d were based on the mean pre-post change in the intervention group minus the mean pre-post change in the control group, divided by the pooled pre-test standard deviation (for details, see Morris, 2008):

$$d=c_{p}\left[\frac{{\left(M_{post,I}-M_{pre,I}\right)-M}_{post,C}-M_{pre,C}}{SD_{pre}}\;\right]$$

where $SD_{pre}$ denotes the pooled standard deviation and $c_{p}$ denotes the bias adjustment for sample size (Becker, 1988). Similarly, follow-up calculations of effect sizes were based on the mean pre-follow-up change in the intervention group, minus the mean pre-follow-up change in the control group, divided by the pooled pre-test standard deviation.

As this effect size requires pre- and post-treatment outcome means, standard deviations, and sample sizes for intervention and control groups, only studies that reported these were included in effect size calculations. For studies with missing information, we contacted the corresponding authors to obtain summary statistics. One of the authors provided this information (Chan and Neece, 2018). Thus, effect sizes of parental well-being were calculated for pre-post-treatment effects in 16 studies, and for follow-up effects for six studies. Effect sizes of child behavior were calculated for pre-post treatment effects in 12 studies, and for follow-up effects for four studies.

Effect sizes were considered to be small when d < 0.5, medium at 0.5–0.79, and large when d ≥ 0.8 (Cohen, 1992).

### Risk of bias

Authors KR, SP and JV assessed the risk of bias in the randomized trials using the Cochrane Collaboration’s tool (Higgins et al., 2011). Selection bias was assessed for aspects of random sequence generation and allocation concealment (Schulz et al., 2018). Performance bias was assessed for blinding of participants and personnel. Detection bias was assessed for blinding of outcome assessment. Attrition bias was assessed for incomplete outcome data. Reporting bias was assessed for selective reporting. Agreement was reached through discussions.

## Results

### Study selection

**Figure 1.**
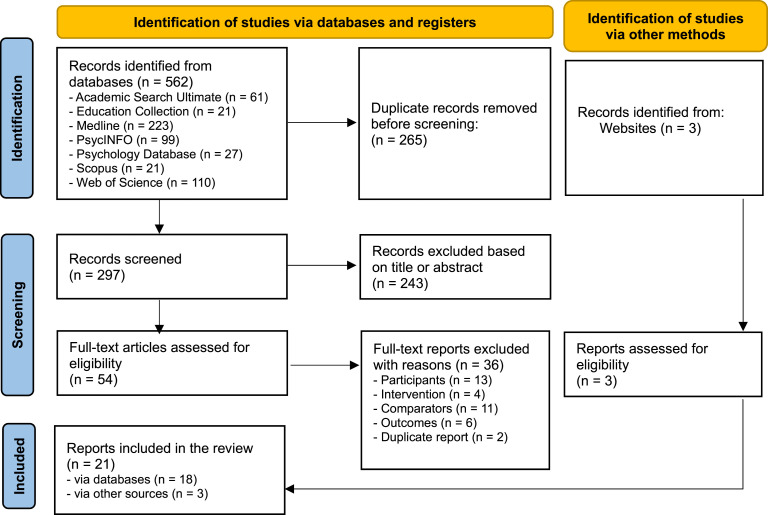
PRISMA flow diagram.

The systematic search in seven databases yielded a total of 562 studies. Duplicates were removed using Zotero 6.0.22. The titles and abstracts of the remaining 297 studies were screened, after which 54 studies remained for full-text screening. Thirty-six studies were excluded, mainly because parents of children with intellectual disabilities were not reported as participants, or there was no comparison group. Eighteen full-text screened studies were included in the review. Manual website searches yielded three additional studies. Twenty-one studies, one of which was a follow-up study, were included in this review. A PRISMA flow chart (Page et al., 2021) describing the identification process is presented in Figure 1.

### Study characteristics

Twenty-one studies were reviewed. They were based on 20 original interventions, and one paper was a follow-up study (Hall et al., 2022).

Of the 21 studies included in the review, 16 studies were reports of original randomized controlled trials (RCT) (Chan and Neece, 2018; Coulman et al., 2022; Grenier-Martin et al., 2022; Hall et al., 2020; Hamdani et al., 2021; Hand et al., 2013; Hinton et al., 2017; Kleefman et al., 2014; Kostulski et al., 2021; Lee et al., 2022; López-Liria et al., 2020; Platje et al., 2018; Roux et al., 2013; Shapiro et al., 2014; Sofronoff et al., 2011; Yıldırım et al., 2012). Four study designs were quasi-experimental (QED) (Ashori et al., 2019; Faramarzi and Bavali, 2017; Kulbaş and Özabacı, 2022; Riemersma et al., 2022). One paper was a follow-up study (Hall et al., 2022) of an included randomized controlled trial (Hall et al., 2020).

Three studies were conducted in Australia, three in the Netherlands, three in the USA, two in Iran, and two in Turkey. One study was conducted in each of the following countries: Canada, Germany, Ireland, Korea/New Zealand, Pakistan, Spain, and the UK.

A total of 1825 caregivers were reported as participants in the 20 studies based on original interventions. Sample sizes ranged from 26 to 540 participants.

### Population characteristics

The gender of the participating caregivers was reported in 17 studies of original interventions. In those studies, 1339 (86.5%) of the 1548 caregivers were female, mostly biological mothers, but including also adoptive foster and stepmothers, grandmothers, aunts, and sisters. Male caregivers were a minority of 13.5% in the gender-reporting studies.

Eight studies reported that all their participants were caregivers of children with intellectual disabilities. The remaining studies also reported child diagnoses in addition to intellectual disabilities, such as autism spectrum disorders, borderline functioning, brain injury, developmental and physical disabilities, or genetic conditions.

The child age ranged from 1.5 to 20 years. In most of the studies, the mean age was under ten years. In three studies, the child mean age was over ten years, with the highest reported mean age being 15.48 years. Study and population characteristics are presented in Table 1.

**Table 1.** Study and Population Characteristics.

Authors	Country	Study design	Sample size (intervention/ control)	Child diagnosis	Child age range, years (mean)
Ashori et al. (2019)	Iran	QED	36 (18/18)	Mild intellectual disabilities (IQ 60–70)	7–10 (8.47)
Chan and Neece (2018)	USA	RCT	80 (39/41)	Developmental delays, autism spectrum disorders, mild-to-moderate intellectual disabilities	2.5–5 (4.18)
Coulman et al. (2022)	UK	RCT	95 parents (48/47);	Any severity of intellectual disabilities	1.5–5
			74 families (37/37)		
Faramarzi and Bavali (2017)	Iran	QED	26 (13/13)	Mild intellectual disabilities (IQ 50–75)	school age
Grenier-Martin et al. (2022)	Canada	RCT	42 (22/20)	Intellectual and developmental disabilities	1.9–7.8 (4.1)
Hall et al. (2020 and 2022)	USA	RCT	60 (32/28); follow-up 34 families (16/18)	FragileX	3.2–10.7 (6.8)
Hamdani et al. (2021)	Pakistan	Cluster RCT	540 parents, 30 clusters (15/15)	Developmental delays and disorders such as intellectual disabilities and Down syndrome	(6.72)
Hand et al. (2013)	Ireland	RCT	29 (16/13)	Mild intellectual disabilities	2–12
Hinton et al. (2017)	Australia	RCT	98 (51/47)	Developmental, intellectual and physical disabilities	2–12 (6.01)
Kleefman et al. (2014)	Netherlands	RCT	209 (111/98)	Borderline (IQ 70 to 85) or mild intellectual disabilities (IQ 70 to 50)	5–12 (9.91)
Kostulski et al. (2021)	Germany	RCT	42 (21/21)	Moderate or mild intellectual disabilities	6–16 (11.19)
Kulbaş and Özabacı (2022)	Turkey	QED	39 (13=intervention, 13=placebo, 13=control)	Moderate or severe intellectual disabilities	school age
Lee et al. (2022)	Korea / New Zealand	RCT	38 (21/17)	Developmental disabilities including autism spectrum disorders, intellectual disabilities, Down Syndrome, and developmental delay	2–10 (5.87)
López-Liria et al. (2020)	Spain	RCT	100 (50/50)	Intellectual disabilities or autism spectrum disorders, sensory disabilities	(9.7)
Platje et al. (2018)	Netherlands	RCT	86 (44/42)	Visual or visual-and-intellectual disabilities	1–5 (3.36)
Riemersma et al. (2022)	Netherlands	QED	41 parents (24/17)	Mild intellectual disabilities	10–20 (13.79 in IG)
Roux et al. (2013)	Australia	RCT	55 (28/27)	Autism spectrum disorders, Intellectual disabilities, Down syndrome, Cerebral Palsy	2–9 (4.8)
Shapiro et al. (2014)	USA	RCT	49 (25/24)	Low birth weight, Down Syndrome, genetic conditions, developmental delays, elevated future risk	0–2 (1.6)
Sofronoff et al. (2011)	Australia	RCT	70 (35/35)	Autism spectrum disorders, intellectual disabilities, developmental delay, acquired brain injury, other disorders	2–10 (6.15)
Yıldırım et al. (2012)	Turkey	RCT	90 (45/45)	Intellectual disabilities (IQ 25 to 50)	(15.48)

Abbreviations: QED = Quasi-experimental design; RCT = Randomized controlled trial.

### Intervention characteristics

Intervention characteristics and contents are summarized in Table 2 and the content themes in Table 3.

**Table 2.** Intervention Characteristics.

Authors (year)	Intervention name	Mode	Sessions and schedule	Theory or framing	Intervention content in brief	Content themes			
						1. Child behavior	2. Interaction and learning	3. Understanding disability	4. Parental well-being
Ashori et al. (2019)	Triple-P	Group	12 weekly 80-min sessions	Social learning theory; Child and family behavior therapy; Applied behavior analysis; Social information processing models; Risk and protective factors (Sanders et al., 2022 and 2004)	Triple P principles: ensuring a safe, interesting environment; creating a positive learning environment; using assertive discipline; having realistic expectations; and taking care of oneself as a parent. SSTP includes additional principles related to disability: family adaptation to having a child with a disability and being part of the community (Sanders et al., 2022 and 2004). SSTP provides various levels and modes of support according to parents' needs. Strategies used in SSTP interventions are described by Sanders et al. (2004).	x	x		x
Hinton et al. (2017)	Triple P Online – Disability (TPOL-D)	Self-directed via internet	8 weekly self-directed modules			x	x	x	
Kleefman et al. (2014)	Stepping Stones Triple P (SSTP)	Individual	8–10 sessions for 40–90 min, over a period of 10–12 weeks			x	x	x	x
Lee et al. (2022)	SSTP Level 2	Group	3 seminars for 2 hours			x	x	x	x
Roux et al. (2013)	SSTP	Group	6 group sessions for 2–2.5 h, 3 individual telephone sessions for 15–30 min			x	x		
Shapiro et al. (2014)	SSTP Level 4	Home visits	10 sessions over 5 months			x	x		
Sofronoff et al. (2011)	SSTP	Group	2 seminars for 2 h			x	x	x	x
Chan and Neece (2018)	Mindful Awareness for Parenting Stress (MAPS)	Group	8 weekly 2-hour sessions and a 6-hour meditation retreat, daily home practice	Mindfulness-based stress reduction (MBSR)	1 Concept of mindfulness 2 Psychology and physiology of stress and anxiety 3 Mindfulness in everyday life in response to challenges and distress				x
Coulman et al. (2022)	Early Positive Approaches to Support (E-PAtS)	Group	8 weekly 2–2.5-h sessions	Research evidence in ID, early intervention theory, developmental systems model, and principles of co-production	1 Working together; e.g. orientation, access to support services 2 Looking after you and your family; e.g. well-being, emotional coping strategies 3 Supporting sleep 4 Interaction and communication; e.g. receptive and expressive communication 5 Supporting active development; e.g. activity engagement and skill development 6 Supporting challenges; e.g. reducing risk of behaviors that challenge 7 Supporting challenges; e.g. reactive and proactive behavioral supports 8 Bringing it all together; e.g. future plans	x	x		x
Faramarzi and Bavali (2017)	Group Logotherapy	Group	8 weekly 90-min sessions	Logotherapy	1 Introducing and familiarizing 2 Assumptions and opinions about life; positive/negative thoughts and feelings 3 Components of meaningfulness in life; purposefulness, values, efficacy and usefulness, self-worth 4 Purposefulness 5 Values 6 Self-efficacy 7 Self-worth 8 Summarizing				x
Grenier-Martin et al. (2022)	Online Training to Support Caregivers of Young Children with Intellectual and Developmental Disability (ABA Online)	Online at parents own pace	5 modules, total ca. 4 hours	Applied behavior analysis (ABA)	1 Documenting and assessing functions of problem behaviors 2 Intervention strategies of antecedents (i.e., prevention) 3 Influence of consequences on behavior, reactions to problem behaviors 4 Appropriate behavior as an alternative to problem behavior 5 Summarizing	x			
Hall et al. (2020 and 2022)	Functional communication training (FCT)	Individually via Zoom	37 sessions median (range 2 to 47) for 1 h over a period of 12 weeks, follow-up booster session	Functional communication training (FCT) / ABA	1 Identifying potential social reinforcers maintaining challenging behavior 2 Teaching an alternative functional communication response to gain reinforcement more appropriately	x	x		
Hamdani et al. (2021)	Technology-assisted, family volunteer-led, parents’ skills training (PST)	Group	Fortnightly sessions over a period of 4 months	Psychoeducation guided by World Health Organization Mental Health Gap Intervention Guide	1 Developmental disorders; e.g. strategies to engage and promote child development 2 Causes and prevention of developmental disorders 3 Play; e.g. caregiver-child interaction 4 Communication; e.g. promoting child’s communication 5 Managing challenging behaviors; e.g. signals before challenging behavior, praise and encouragement, teaching words and gestures to ask, picture schedules 6 Managing everyday life situations 7: Managing stress 8: Comorbid conditions; e.g. epilepsy, depression, ADHD; Promoting community-based rehabilitation and human rights 9 Motor difficulties	x	x	x	x
Hand et al. (2013)	Parents Plus Children’s Programme (PPCP)	Group	8 weekly 2.5-h sessions	Solution-focused systemic therapy, social learning theory, parent training, cognitive behavior therapy, conflict management and negotiation theory, and developmental psychology (Carr, 2017)	1 Solving children’s problems; Dealing with special needs 2 Positive instruction; Importance of play and special time 3 Establishing routines; Best way to play 4 Using consequences; Power of encouragement 5 Supporting homework and using sanction system; Encouraging self-esteem 6 Assertive parenting/dealing with disrespect; Prevention plans 7 Problem solving with children 8 Topics chosen by families, Review	x	x	x	
Kostulski et al. (2021)	Parent management training (PMT)	Group	10 sessions for 90 min, over a period of 6 months	Psychoeducation developed based on PMT for attention-deficit/hyperactivity disorder and autism spectrum disorders	1 Information about intellectual disabilities and specific diagnosis; accepting child’s intellectual disabilities 2 Improving parent management skills in problematic situations 3 Reducing the burden on the family and provintellectual disabilitiesing information about helpful services 4 Fostering positive aspects of the child	x		x	x
Kulbaş and Özabacı (2022)	Positive Psychology-Based Online Group Counselling Program (PPBOGCP)	Online Group	60–90 min sessions over a period of 10 weeks	Multidimensional Psychological Well-Being Model, Awareness-Based Self-Compassion Programs, Hope Theory	1 Construction (of the group) 2 Strengths (of group members) 3 Self-acceptance and acceptance of the past 4 Getting to know feelings 5 Self-compassion (free from self-judgmental criticism, mindfulness skills) 6 Relationships with others (relationships/support mechanisms) 7 Thanksgiving (positive things they have) 8 Optimism (healing power of optimism) 9 Hopeful life (goals and motivation to take action) 10 Saying goodbye and ending				x
López-Liria et al. (2020)	Training Program in the Management of Stress for Parents of Disabled Children (TPMS)	Group	7 weekly 90-min sessions	Stress appraisal and coping model	1 Training program presentation; Stress conceptualization 2 Coping strategies; Relaxation training; Control of breathing 3 Cognitive restructuring of a sense of guilt; Training of coping skills, relaxation and breathing 4 Problem-solving; Rewarding activities; Skills acquisition and practice 5 Increase in rewarding activities; Self-esteem; Problem-solving 6 Assertive communication; Humor and optimism; Social support; Breathing and relaxation 7 Review				x
Platje et al. (2018)	Video-feedback Intervention to promote positive parenting-visual (VIPP-V)	Home visits	7 home-visits for 1.5 h: 5 every 2–3 weeks, 2 in next 2 months	Social learning and attachment theory	1 Exploration versus attachment behavior; when and how child needs their parent 2 Speaking for the child; verbalizing the child’s nonverbal cues 3 Sensitivity chain; parents’ sensitive responses, child’s positive reaction 4 Sharing emotions; affective attunement to child’s emotions		x		
Riemersma et al. (2022)	‘You are Okay’	Online program with home-visits for parents, group for children	Parents 3 sessions; Children 10 weekly 1.5-h sessions and a booster after 6 weeks	Empirical knowledge about risk and protective factors	Program for parents: 1 Negative cognitions; confidence for being a good parent 2 Possible influence of parents' mental health concerns on the children 3 Communication; understanding children, discussing mental health concerns 4 Positive behavior of the children; compliments and boundaries 5 Social network; social support. **Support group for children:** 1 Get to know each other 2 Recognize problems at home 3 Recognize basic emotions 4 Recognize emotions in difficult situations 5 Show emotions 6 Understand mental health concerns 7 Use social network 8 Cope with difficult situations 9 Develop social skills 10 Parting session 11 Booster session	x	x		x
Yıldırım et al. (2012)	Psychosocial nursing education (PNE)	Group	4 weekly 2-h sessions	Social learning theory, communication skills and coping with stress	1 Characteristics, education, care of intellectually disabled children, positive features of the child 2 Communication techniques, communication in the family, coping with stress 3 Effective problem-solving methods, responding to depressive situations 4 Discussion, other support to children and families, legal matters		x	x	x

**Table 3.** Intervention Content Themes and Examples.

General Content Themes	Content Themes and Subthemes	Examples
**Child or Interaction**	**Child Behavior**	
	Preventing challenging behavior	Identifying causes, signals, and functions of challenging behavior
		Positive reinforcement, Praise and encouragement, Rewards
		Positive attention
		Teaching appropriate behavior
		Giving a good example
		Rules and routines
		Clear and calm instructions
	Reacting to challenging behavior	Withdrawal of attention, Ignoring minor problem behavior
		Blocking dangerous behavior
		Assertive discipline
		Logical consequences
	**Interaction and Learning**	
	Parent-child interaction	Caregiver-child interaction
		Play and special time
		Showing affection, Sharing emotions
		Parent's sensitive responses
		Understanding children
		Problem solving with children
	Communication	Communication in the family
		Communication techniques (e.g. pictures)
		Teaching alternative functional communication
	Child learning	Creating positive learning environment
		Having realistic expectations
		Supporting homework, Supporting active development
		Supporting child sleep
	**Understanding Disability**	Information about intellectual disabilities
		Family adaptation to having a child with a disability
		Care of intellectually disabled children
		Comorbid conditions; epilepsy, depression, attention-deficit/ hyperactivity disorder, motor difficulties
		Positive aspects of a disabled child
		Human rights of a disabled child
**Parent**	**Parental Well-being**	Taking care of oneself as a parent
		Coping with stress
		Emotional coping
		Meaningfulness in life, Values
		Self-compassion, Self-efficacy, Self-worth
		Mindfulness, Relaxation, and breathing
		Humor and optimism
		Parental sleep
		Social support, Support services

The interventions had various theoretical foundations. Seven of the 20 interventions were based on Triple P, or Stepping Stones Triple P (Ashori et al., 2019; Hinton et al., 2017; Kleefman et al., 2014; Lee et al., 2022; Roux et al., 2013; Shapiro et al., 2014; Sofronoff et al., 2011). Triple P – Positive Parenting Program is an Australian-based system of interventions for parents of children who have, or are at risk of having, behavioral or emotional problems. Stepping Stones Triple P (SSTP) was designed for families who have a child with a disability (Sanders et al., 2004). Triple P and SSTP are based on social learning theory; child and family behavior therapy; applied behavior analysis (ABA); social information processing models; as well as risk and protective factors (Sanders et al., 2022 and 2004). In Table 2, Triple P/SSTP interventions are synthesized by the program’s common principles.

In addition, three interventions were based on social learning theory (Hand et al., 2013; Platje et al., 2018; Yıldırım et al., 2012). The Parents Plus Children’s Programme (PPCP) in Ireland had foundations in social learning theory, solution-focused systemic therapy, parent training, cognitive behavior therapy (CBT), conflict management and negotiation theory, and developmental psychology (Hand et al., 2013; Carr et al., 2017). Video-feedback Intervention-visual (VIPP-V) in the Netherlands was based on social learning and attachment theories (Platje et al., 2018; Van IJzendoorn et al., 2023). Psychosocial nursing education (PNE) in Turkey was based on social learning theory, communication skills and coping with stress (Yıldırım et al., 2012).

In addition to Triple P/SSTP, two interventions were based on ABA: Online Training to Support Caregivers of Young Children with Intellectual and Developmental Disability (ABA Online) in Canada (Grenier-Martin et al., 2022); and Functional communication training (FCT) in the US (Hall et al., 2020 and 2022).

Two interventions were reported as psychoeducational. The largest intervention, Technology-assisted, family volunteer-led, parents’ skills training (PST) in Pakistan was psychoeducational and based on the World Health Organization Mental Health Gap Intervention Guide (Hamdani et al., 2021). Parent management training (PMT) was a psychoeducation program in Germany (Kostulski et al., 2021). Its development was based on parent management training for attention-deficit/hyperactivity disorder and autism spectrum disorders.

Early Positive Approaches to Support (E-PAtS) intervention in the UK was based on research evidence in intellectual disabilities, early intervention theory, developmental systems model, and principles of co-production (Coulman et al., 2022). ‘You are Okay’ intervention in the Netherlands was based on empirical knowledge about risk and protective factors (Riemersma et al., 2022).

Group logotherapy in Iran (Faramarzi and Bavali, 2017) was based on logotherapy to reach the meaning of life. The Positive Psychology-Based Online Group Counselling Program (PPBOGCP) in Turkey was based on a multidimensional psychological well-being model, self-compassion programs and hope theory (Kulbaş and Özabacı, 2022). The Training Program in the Management of Stress for Parents of Disabled Children (TPMS) in Spain was based on a stress appraisal and coping model (López-Liria et al., 2020). Mindful Awareness for Parenting Stress (MAPS) was a mindfulness-based stress reduction program in the US (Chan and Neece, 2018).

Thirteen of the 20 interventions were delivered in a group format, one of which was an online group (Kulbaş and Özabacı, 2022). Two interventions were provided as home-visits (Platje et al., 2018; Shapiro et al., 2014). One study reported individual sessions but not the setting (Kleefman et al., 2014). One program was delivered individually via Zoom to parents with their children (Hall et al., 2020 and 2022). Two interventions were self-directed by parents via the internet (Hinton et al., 2017; Grenier-Martin et al., 2022). One program included a support group for children, and an online program for parents with mental health concerns, with a social worker visiting the parents (Riemersma et al., 2022).

Twelve studies reported that program providers were professionals such as psychologists, health and social care, education, or third-sector specialists. The largest intervention was delivered by trained family volunteers in Pakistan. Professionals and trained family carers co-delivered a program in the UK. Two online programs were self-directed by parents.

The shortest interventions totaled four to six hours (Grenier-Martin et al., 2022; Lee et al., 2022; Sofronoff et al., 2011). Ten of the interventions included seven to ten sessions of 40 minutes to 2.5 hours over a period of seven weeks to six months. The largest number of sessions was provided in FCT; the median number of FCT sessions was 37 over a period of 12 weeks (Hall et al., 2020).

#### Intervention content analysis

In a content analysis of the interventions, two general content themes and four content themes with their subthemes emerged. These themes were:

**Child or interaction**

1 Child behavior

2 Interaction and learning

3 Understanding disability

**Parent**

4 Parental well-being

The general content theme ‘Child or interaction’ included three content themes addressing the child or interaction with the child. The content theme 1 ‘Child behavior’ included subthemes of preventing and reacting to the child’s behavioral challenges. Strategies such as positive reinforcement andihdrawal of attention were used. Theme 2, ‘Interaction and learning’, included subthemes of parent–child interaction, communication, and child learning. In addition to parental sensitive attendance and joint activities with children, communication and supporting child learning were considered to be parts of the theme. This theme included play and special time, communication in the family, and supporting active development, for example. The theme overlapped extensively with preventing and reacting to challenging behaviors. Theme 3, ‘Understanding disability’, covered information about the child’s disability and seeing positive aspects of the child.

The general content theme ‘Parent’ included theme 4, ‘Parental well-being’. Content in this theme directly targeted parental stress, coping and well-being, for example, by relaxation and breathing, meaningfulness, self-compassion, and optimism. The themes and examples of intervention contents are presented in Table 3.

The 20 original interventions were categorized by their content. The same intervention addressed multiple types of content. Nine interventions reported content on both general themes ‘Child or interaction’ and ‘Parent’ (Ashori et al., 2019; Coulman et al., 2022; Hamdani et al., 2021; Kleefman et al., 2014; Kostulski et al., 2021; Lee et al., 2022; Riemersma et al., 2022; Sofronoff et al., 2011; Yıldırım et al., 2012). Four of these nine interventions reported including all four content themes: 1 Child behavior; 2 Interaction and learning; 3 Understanding disability; and 4 Parental well-being. One of these was the largest intervention, PST, which targeted at promoting children’s communication, socioemotional development and adaptive behaviors; managing co-morbid conditions and motor difficulties; and improving the psychological well-being of caregivers in Pakistan (Hamdani et al., 2021). The other three interventions were based on Stepping Stones Triple P in the Netherlands (Kleefman et al., 2014), in Korea/New Zealand (Lee et al., 2022), and in Australia (Sofronoff et al., 2011). Triple P principles include ensuring a safe, interesting environment; creating a positive learning environment; using assertive discipline; having realistic expectations; and taking care of oneself as a parent. SSTP includes additional principles related to disability: family adaptation to having a child with a disability and being part of the community (Sanders et al., 2022 and 2004). However, all the Triple P/SSTP interventions did not report including all the content themes which emerged in this review. Five studies reported content on three themes relating to the general themes ‘Child or interaction’ and ‘Parent’. A Triple P intervention in Iran covered child behavior, child development, and survival and stability of the family (Ashori et al., 2019). The E-PAtS program in the UK included parental well-being; interaction, communication, and development, as well as child behavior (Coulman et al., 2022). PMT in Germany contained information about intellectual disabilities, child behavior, and reducing the burden on the family (Kostulski et al., 2021). The ‘You are Okay’ program for parents included child behavior, communication, and support for the parents in the Netherlands (Riemersma et al., 2022). PNE in Turkey contained information about care of intellectually disabled children, communication, and stress (Yıldırım et al., 2012).

Seven interventions focused on the general content theme ‘Child or interaction’ (Hall et al., 2020 and 2022; Hand et al., 2013; Hinton et al., 2017; Roux et al., 2013; Shapiro et al., 2014; Grenier-Martin et al., 2022; Platje et al., 2018). Two studies reported content on three themes related to ‘Child or interaction’. Triple P Online-D included content on child behavior, learning and disability in Australia (Hinton et al., 2017). The PPCP intervention in Ireland dealt with child behavior, learning, and special needs (Hand et al., 2013). Three interventions covered two content themes. SSTP interventions reported content on child behavior and learning in Australia (Roux et al., 2013) and in the US (Shapiro et al., 2014). FCT in the US covered child behavior and appropriate forms of communication (Hall et al., 2020 and 2022). Two interventions focused on a single content theme addressing ‘Child or interaction’. ABA Online in Canada covered child behavior (Grenier-Martin et al., 2022). VIPP-V focused on interaction with the child in the Netherlands (Platje et al., 2018).

Four interventions focused on the general theme ‘Parent’ and parental well-being (Chan and Neece, 2018; Faramarzi and Bavali, 2017; Kulbaş and Özabacı, 2022; López-Liria et al., 2020). MAPS covered mindfulness-based stress reduction in the US (Chan and Neece, 2018). Group logotherapy addressed components of the meaningfulness of life in Iran (Faramarzi and Bavali, 2017). PPBOGCP in Turkey included self-acceptance, self-compassion, optimism, and hope (Kulbaş and Özabacı, 2022). TPMS in Spain contained stress coping, relaxation and breathing (López-Liria et al., 2020).

### Comparison group characteristics

According to the eligibility criteria, studies were included in this review if the control groups comprised parents receiving no treatment, treatment as usual (TAU), or on waiting list. Six studies reported that comparison groups received treatment or care as usual. In one study, TAU was enhanced. Hence, community health workers received training in recognizing developmental disorders.

Seven studies reported allocating parents to waitlist control groups or optional waitlist groups. One program provided a shorter version of the intervention program to the control group at the end of the research. One study allocated participants to experimental, placebo, and control groups. The placebo group was not included in this review.

### Results of individual studies

Results of the studies and effect sizes calculated in this review are shown in Table 4.

**Table 4.** Results of Studies and Calculated Effect Sizes.*

Authors	Results of studies	Parent well-being measures	Pre-post intervention-control effect size Parent (Morris 2007)	Follow-up effect size Parent (Morris 2007)	Child behavior measures	Pre-post intervention-control effect size Child (Morris 2007)	Follow-up effect size Child (Morris 2007)	Time of measurement T2	Follow-up period (after T2)
Ashori et al. (2019)	Improvements in somatic symptoms, anxiety, depression, social dysfunction, and mental health.	GHQ Total mental health	**3.88**					Post-test	
Chan and Neece (2018)	Improvements in mental health outcomes, and child attention and withdrawn behaviors, maintained at follow-up.	PSI–Short Form Parental Distress Subscale	**1.11**		CBCL (1.5–5) Aggressive Behavior	0.06		Post-test	6 months
		CES-D Total Depression Score	**0.97**		CBCL (1.5–5) Attention Problems	0.55			
		SWL Total Score	0.47						
Coulman et al. (2022)	Well-being score was higher at 12 months post randomization.	WEMWBS	0.22	0.49	CBCL Total problems	0.24	0.41	One month post test	9 months
		HADS Emotional distress: sum of anxiety and depression subscales	0.21	0.24					
Faramarzi and Bavali (2017)	Increased psychological well-being including positive relationships with others, autonomy, personal growth, and environmental mastery.	RSPWB Total score	0.40					Post-test	
Grenier-Martin et al. (2022)	Reductions in the frequency of problem behaviors and parenting stress, and improvements in caregivers’ self-efficacy.	PSI 36-item; PSOC	n.a.		ABAS–2nd Edition; BPI–01	n.a.		One month post test	2 months
Hall et al. (2020 and 2022)	Irritability, problem behaviors and parenting stress decreased. Improvements in the irritability and lethargy/social withdrawal subscales over the follow-up interval.	PSI, 4th Edition 120-item	n.a.		ABC – Community; BPI – Short Form	n.a.		Post-test	2.8 years for IG; 3.2 years for TAU on average
Hamdani et al. (2021)	Improved parental health related quality of life.	Peds-QL Family impact module Total score	0.30		SDQ Total socio-emotional difficulties	0.13		6 months post test	
Hand et al. (2013)	Reductions in child problem behavior and parent stress.	PSI 36-item total	0.67		SDQ Total difficulties	**1.23**		Post-test	
Hinton et al. (2017)	Improvements in parenting self-efficacy. Decrease in child behavioral and emotional problems at follow-up.	CAPES-DD Self-Efficacy	0.75		DBC – Primary Carer version Total	0.23		Post-test	3 months
					CAPES-DD Total Problems scale	0.03			
Kleefman et al. (2014)	For parenting stress and a child’s psychosocial problems at school, advantages were found in the short term. No differences between groups were found in the longer term.	PSI 25-item	0.29	0.12	SDQ Parent version	n.e.	n.e.	Post-test	6 months
					SDQ Teacher version	0.23	0.17		
					ECBI	n.e.	n.e.		
Kostulski et al. (2021)	Reduction of behavioral and emotional problems and family burden.	IFS (FaBel) total	0.63		DBC (VFE) total	0.76		Post-test	
Kulbaş and Özabacı (2022)	Increase in psychological well-being, self-compassion and hope levels.	RSPWB (PWBS) total	**7.46**	**7.28**				Post-test	2 months
		DHS total	**3.37**	**5.65**					
		SCS total	**5.65**	**3.39**					
Lee et al. (2022)	Reductions in child behavior and emotional difficulties were maintained 4 months later.	CAPES-DD Self-efficacy	n.a.		DBC-24 Short version	0.70		6 weeks post test	4 months
					CAPES-DD Behavioral problems	0.67			
López-Liria et al. (2020)	Improvements in parents' emotional states, stress levels, and adjustment at post-test and follow-up.	PSI 36-item	0.69	0.78				Post-test	6 months
		MAS Total	0.77	0.57					
Platje et al. (2018)	Decrease in parenting stress. Higher parenting self-efficacy at follow-up.	SNR Total	0.09	0.35				7 weeks post test on average	6 months on average
		PSI 25-item	0.35	0.22					
Riemersma et al. (2022)	Parents reported lower levels of child difficulties. Children reported decrease in emotional and behavioral problems.	PSAM; PSI – Short Form	n.a.		SDQ Child report Total difficulties	0.41	**0.96**	Post-test	4 months
					SDQ Parent report Total difficulties	0.31	0.70		
Roux et al. (2013)	Improvements in parental psychological functioning. Improvements in child behavior were maintained at follow-up.	DASS total	0.54		ECBI Problem scale	**0.82**		Post-test	6 months
					DBC – Parent version Total	0.72			
Shapiro et al. (2014)	No between-group differences were seen aside from reduced parental depression at follow-up.	DASS-21 Depression	0.16	0.40	CBCL (1.5–5) Total	0.07	0.03	Post-test	12 months
Sofronoff et al. (2011)	Reductions in child behavior problems were maintained at follow-up. Sleeper effect for parenting confidence.	DASS-42 Stress	0.34		ECBI Problem scale	0.61		6 weeks post test	3 months
		PSOC Efficacy	0.04						
Yıldırım et al. (2012)	Decrease in the risk for depression.	BDS Total	0.64					One month post test	

* Effect sizes are reported as absolute values.

Abbreviations: ABC = Aberrant Behavior Checklist; ABAS = Adaptive Behavior Scale Assessment System; BDS = Beck Depression Scale; BPI = Behavior Problems Inventory; CAPES-DD = Child Adjustment and Parent Efficacy Scale – Developmental Disability; CBCL = Child Behavior Checklist; CES-D = Center for Epidemiologic Studies Depression Scale; DASS = Depression Anxiety Stress Scales; DBC = Developmental Behavior Checklist; DHS = Dispositional Hope Scale; ECBI = Eyberg Child Behavior Inventory; GHQ = General Health Questionnaire; HADS = Hospital Anxiety and Depression Scale; IFS = Impact on Family Scale; MAS = Maladjustment scale; Peds-QL = Pediatric Quality of Life; PSAM = Parenting Self-Agency Measures; PSI = Parenting Stress Index; PSOC = Parenting Sense of Competence; RSPWB = Ryff’s Scale of psychological well-being; SCS = Self-Compassion Scale; SDQ = Strengths and Difficulties; SNR = Self-efficacy in the Nurturing Role; SWL = Satisfaction with Life; WEMWBS = Warwick–Edinburgh Mental Well-Being Scale Questionnaire.

n.a. = not available; n.e. = no effect over the control group: improvements were stronger in the control group than in the intervention group.

#### Short-term effects on parental well-being

We first investigated the short-term effects of interventions by comparing pre- and post-treatment scores in intervention and control groups. Very large effect sizes (above 3.0) of parental well-being were found for two interventions: the PPBOGCP program, which was based on a multidimensional psychological well-being model, self-compassion programs and hope theory (Kulbaş and Özabacı, 2022); and Triple P (Ashori et al., 2019). These studies were limited by quasi-experimental designs and small sample sizes.

Large to small effect sizes (1.11 to 0.47) for parental outcomes were found in a mindfulness-based MAPS program (Chan and Neece, 2018). Medium effect sizes (range 0.54 to 0.77) were found for six RCTs. These interventions were a TPMS program which was based on stress appraisal and coping model (López-Liria et al., 2020); Triple P Online – Disability (Hinton et al., 2017); PPCP program based on solution-focused systemic therapy, social learning theory, and cognitive behavior therapy (Hand et al., 2013); PNE nursing education based on social learning theory, communication skills and coping with stress (Yıldırım et al., 2012); PMT psychoeducation (Kostulski et al., 2021); and SSTP (Roux et al., 2013).

Small (below 0.5) effect sizes were calculated for an additional seven studies consisting of six RCTs and one QED. These included two largest studies: PST psychoeducation (Hamdani et al., 2021); and SSTP (Kleefman et al., 2014).

The nine interventions which produced medium to very large effect sizes (range 0.5 to 7.5) of improvements in parental well-being were delivered to caregivers of children aged two years or older. Eight of these interventions were delivered in a group or an online group format by professionals, and one intervention with a medium effect size was self-directed by parents via the internet. Five of these nine interventions included only parents of children with intellectual disabilities. Eight of these nine programs provided six to 12 sessions for 60 minutes to 2.5 hours each. One program with a medium effect size on parental well-being provided four sessions, each of 2 hours (Yıldırım et al., 2012).

Measures of positive parental well-being produced the largest effect sizes. Medium effect sizes were found for improvements in both psychological illness and psychological well-being.

#### Short term effects on child behavior

Large effect sizes on child behavior (range 0.82 to 1.23) were found for the PPCP program based on solution-focused systemic therapy, social learning theory and CBT (Hand et al., 2013), and SSTP (Roux et al., 2013). Both studies were RCTs with small sample sizes. Four RCTs produced medium effect sizes (range 0.55 to 0.72) on child behavior, two of which were based on SSTP (Lee et al., 2022; Sofronoff et al. 2011), one was based on PMT psychoeducation (Kostulski et al., 2021), and one on MAPS mindfulness intervention (Chan and Neece, 2018). MAPS produced a medium effect on child attention problems, and a very small effect on aggressive behavior. Small effect sizes on child behavior (below 0.5) were calculated for six studies consisting of five RCTs and one QED. These included the two largest studies: PST psychoeducation (Hamdani et al., 2021); and SSTP (Kleefman et al., 2014). In the study by Kleefman and colleagues (2014), teacher-reported child behavior produced a small effect size, but parent-reported child behavior did not show improvements over the control group.

All six interventions which produced medium to large effect sizes (range 0.55 to 1.23) of improvements in child behavior were RCTs with small sample sizes of 80 or less participants. They were delivered in a group format by professionals to caregivers of children aged 2 years or older. Two of the six programs were directed to parents of children with mild or moderate intellectual disabilities. Three programs provided 6 to 10 group sessions for 1.5 to 2.5 hours each. Nevertheless, short seminars of 2 to 3 times 2 hours produced medium effect sizes on child behavior at least in the short term (Lee et al., 2022; Sofronoff et al. 2011).

#### Follow-up effects

Follow-up measurements were conducted in 14 of the 21 reviewed studies. Follow-up periods ranged from two months to 2.8 years (on average in Hall et al., 2022). The median reported follow-up period was 6 months. Two studies had follow-up periods of 12 months or more (Hall et al., 2022; Shapiro et al., 2014).

In this review, follow-up effect sizes on parental well-being were calculated for six studies (Table 4) which provided sufficient data. Very large follow-up effect sizes on parental well-being were calculated in the study by Kulbaş and Özabacı (2022); medium effect sizes (range 0.57 to 0.78) in the study by López-Liria et al. (2020); and small effect sizes (range 0.12 to 0.49) for the studies of Coulman et al. (2022); Kleefman et al. (2014); Platje et al. (2018); and Shapiro et al. (2014). Further improvements in effect sizes on parental well-being were found in five studies at follow-up (Coulman et al., 2022; Kulbaş and Özabacı, 2022; López-Liria et al., 2020; Platje et al., 2018; Shapiro et al., 2014).

Follow-up effect sizes on child behavior could be calculated in four studies only (Table 4). A large effect size (0.96) was calculated for child-reported behavior and a medium effect size (0.7) for parent-reported behavior in the study by Riemersma et al. (2022). Small effect size (0.41) on child behavior was calculated in the study by Coulman et al. (2022). Both studies showed further improvements in child behavior at follow-up. Studies by Kleefman et al. (2014) and Shapiro et al. (2014) showed no improvements in child behavior over the control group at follow-up.

In this review, follow-up effect sizes could not be calculated for two studies due to missing information, and for five studies which had no control group at follow-up, because wait-list control groups received intervention before follow-up measurements (Chan and Neece, 2018; Hinton et al., 2017; Lee et al., 2022; Roux et al., 2013; Sofronoff et al., 2011). These five studies reported that most improvements in parental well-being and child behavior were sustained at follow-up. Hinton et al. (2017) and Sofronoff et al. (2011) reported further improvements in parental self-efficacy and child behavior at follow-up.

### Risk of bias and certainty of evidence

The risk of bias chart is shown in Supplemental Figure 1. Eight out of the 16 included original RCTs reported sufficient random sequence generation. Permuted-block designs were considered to cause potential for selection bias (Matts and Lachin, 1988). Five studies reported adequate allocation concealment. Blinding of participants or personnel was typically not possible due to the nature of interventions. Parents were blind to the waitlist-control design in one study. Researchers were reported blind to the allocation status of trial participants in five studies. The blinding of outcome assessors is particularly difficult for participant-reported outcomes (Higgins et al. 2023). All studies used participant reports of outcome measures. Kleefman et al. (2014) used additional teacher reports of child behavior, and Hall et al. (2020) used additional in-session observations. Attrition bias was increased by incomplete outcome data. The median reported drop-out rate of participants in the included studies was 23%. In two studies, the drop-out rate was 49% to 52%, but intention-to-treat analyses were conducted (Kleefman et al., 2014; Shapiro et al., 2014). Concerns about outcome reporting bias arise if studies selectively report outcomes with positive or statistically significant results. Sufficient data for low risk of reporting bias was available in 11 studies.

The results of the studies were heterogeneous, with effect sizes ranging from no effect over the control group, to very large effect sizes. Indirectness of the evidence was decreased by including studies from both high- and low-income settings, but it was increased by short term evaluations (Schünemann et al., 2023). Follow-up measurements were conducted in 14 studies, five of which solely for the intervention group. Only two studies had follow-up periods of 12 months or more. The small number of participants in the studies increased the imprecision of the evidence (Schünemann et al., 2023). Only three studies had one hundred or more participants. Early, positive findings from most interventions were shown, but replicated or partly replicated studies were found for only two programs. We included only studies published in peer-reviewed journals. This may introduce publication bias because published studies are more likely to report positive, or statistically significant findings.

### Discussion

The aim of this review was to identify the type, content, and effectiveness of psychological parenting interventions for parents of children with intellectual disabilities to enhance children’s behavioral outcomes or parental psychological well-being.

### Type and content of interventions

The reviewed interventions had various theoretical foundations. Interventions that addressed the theme ‘Child or interaction’ were based on theories or framings, such as applied behavior analysis (ABA), attachment theory, conflict management and negotiation theory, cognitive behavior therapy (CBT), psychoeducation, risk and protective factors, research evidence in intellectual disabilities, social learning theory, and solution-focused systemic therapy. Interventions that focused solely on the theme ‘Parent’ and parental well-being were based on logotherapy; mindfulness-based stress reduction (MBSR); multidimensional psychological well-being model, self-compassion programs and hope theory; and stress appraisal and coping model.

Previous reviews have studied interventions for parents of children with disabilities or autism spectrum disorders based on ABA, acceptance and commitment therapy (ACT), CBT, psychoeducation, operant and social learning theories, expressive writing, mindfulness, and stress management and relaxation techniques (Bourke;Taylor et al., 2021; Cachia et al., 2016; Chua and Shorey, 2022; Da Paz and Wallander, 2017; Osborn et al., 2021; Ragni et al., 2022; Ruane and Carr, 2019).

None of the previous reviews, or studies in this review, reported that they were based on mentalization (Fonagy and Campbell, 2016), although they may have included mentalizing elements. In the context of parenting, mentalization refers to a parent’s capacity to think about and understand their child’s feelings and experiences. Parent’s higher mentalization capacity is associated with more sensitive parent–child interactions, more secure child attachment, and enhancing child development (Kalland et al., 2016).

To our knowledge, there have been no recent content analyses of interventions for parents of children with intellectual disabilities. Previous studies have analyzed the content of parenting programs for parents of typically developing children and categorized treatment families (Morris et al., 2020; Kaminski and Claussen, 2017). While there were many similarities between Morris and colleagues’ (2020) and our content analysis in child behavior, interaction and learning, our review found more content on parental well-being and understanding disability.

### Effectiveness

#### Effects on parental well-being

All the reviewed studies measured parental well-being. Small effect sizes were calculated for seven studies. Medium to very large effect sizes on parental well-being were produced by nine interventions. Three of these nine programs (MAPS, PPBOGCP, and TPMS) focused on the ‘Parent’ and parental well-being (Chan and Neece, 2018; Kulbaş and Özabacı, 2022; López-Liria et al., 2020). Three interventions (PPCP, and two Triple P/SSTP interventions) focused on the ‘Child or interaction’ (Hand et al., 2013; Hinton et al., 2017; Roux et al., 2013). Another three interventions (PMT, PNE, and Triple P) addressed both ‘Child or interaction’ and ‘Parent’ (Ashori et al., 2019; Kostulski et al., 2021; Yıldırım et al., 2012).

Very large effect sizes of parental well-being were calculated for the PPBOGCP program in Turkey (Kulbaş and Özabacı, 2022); and a Triple P intervention in Iran (Ashori et al., 2019). These studies were conducted in low-income settings in the Middle East and were limited by quasi-experimental designs and small sample sizes. Former evidence suggests that interventions conducted in Middle Eastern countries were more effective in reducing parental distress than programs in Western and Asian countries. It may be easier to make parental well-being trainings a daily routine as part of Islamic religious practices (Chua and Shorey, 2022). However, this does not explain the very large effect size of the Triple P intervention in Iran, which may be explained by other cultural aspects or study limitations, for instance.

At follow-up, parental well-being remained higher than baseline in intervention groups in all studies that reported follow-up results. Further improvements in parental well-being compared to post-test were reported in seven studies at follow-up, indicating a possible sleeper effect (Coulman et al., 2022; Hinton et al., 2017; Kulbaş and Özabacı, 2022; López-Liria et al., 2020; Platje et al., 2018; Shapiro et al., 2014; Sofronoff et al., 2011).

Our findings about parental well-being are consistent with earlier evidence of interventions for parents of children with disabilities or autism spectrum disorders. Previous reviews found that MBSR, ACT, stress management and relaxation techniques, expressive writing, as well as CBT and psychoeducation showed promising results for parental mental health (Bourke;Taylor et al., 2021; Cachia et al., 2016; Chua and Shorey, 2022; Da Paz and Wallander, 2017; Osborn et al., 2021). Masulani-Mwale et al. (2018) found evidence that psychosocial interventions can improve psychological outcomes among the parents of children with intellectual disabilities. On the other hand, Sohmaran and Shorey (2021) found that parental stress was reduced at post-treatment, but not at three to six months post-treatment. In our review, most follow-up studies showed sustained or further increased improvements in parental well-being.

#### Effects on child behavior

Fourteen interventions measured child behavior. Small effect sizes were calculated for six studies. Medium to large effect sizes on improvements in child behavior were produced by six of interventions. Two of these six programs (PPCP and SSTP) focused on the ‘Child or interaction’ (Hand et al., 2013; Roux et al., 2013). Three interventions (PMT, and two SSTP programs) addressed both ‘Child or interaction’ and ‘Parent’ (Kostulski et al., 2021; Lee et al., 2022; Sofronoff et al., 2011). One intervention (MAPS) focused on the ‘Parent’ (Chan and Neece, 2018). Other interventions which focused solely on the ‘Parent’ and parental well-being did not measure effects on child behavior.

At follow-up, four programs produced further improvements in child behavior compared to post-test, indicating a possible sleeper effect. These were E-PAtS; SSTP; Triple P and ‘You are Okay’ (Coulman et al., 2022; Hinton et al., 2017; Riemersma et al., 2022; Sofronoff et al., 2011). Two SSTP studies showed no improvements in child behavior over the control group at follow-up (Kleefman et al., 2014; Shapiro et al., 2014).

Our findings of effective interventions for child behavior are consistent with the results of previous studies of interventions for parents of children with disabilities or autism spectrum disorders. Previous positive results for child behavior were shown by interventions based on the principles of operant and social learning theories and ABA (Ragni et al., 2022); parent-mediated interventions teaching comprehensive skills, or targeting joint attention, communication, or language (Conrad et al., 2021); SSTP (Ruane and Carr, 2019); and mindfulness (Cachia et al., 2016).

#### Other remarks of effectiveness

All 21 studies that we reviewed reported some advantages to parental well-being or child behavior. However, the results of the reviewed studies were heterogeneous, with effect sizes ranging from no effects to very large effects. The results should be interpreted cautiously, because the study designs, small sample sizes, other study limitations, or cultural aspects, may have had an impact on the effect sizes. Studies with small sample sizes produced the largest effect sizes in this review. Small study effects can arise through chance or other sources of heterogeneity, such as methodological differences between studies. Thus, generalizable effects may not be as large as the studies with small sample sizes indicate. The largest studies produced small effect sizes. Results are generally more precise, with large numbers of participants (NHMRC, 2019).

It is worth noting that the results of the seven SSTP/Triple P interventions in this review were heterogeneous, with effect sizes ranging from no effect over the control group to very large effect sizes. All SSTP/Triple P interventions that showed medium to very large effect sizes on parental well-being or child behavior were conducted in Australia, where the program was developed, or in Korea/New Zealand, or in Middle Eastern Iran. These interventions were provided in a group format, except one via an internet self-directed program. Individually provided SSTP interventions in the Netherlands and in the US showed small or no effects over the control group and high drop-out rates (Kleefman et al., 2014; Shapiro et al., 2014).

PPCP intervention (Hand et al. 2013), SSTP (Roux et al., 2013), and PMT (Kostulski et al., 2021) produced medium or large effect sizes on both parental well-being and child behavior. MAPS (Chan and Neece, 2018) produced small to large effects on parental outcomes and small to medium effects on child outcomes.

Promising findings of all the interventions were shown but replicated (Triple P/SSTP) or partly replicated (MAPS) (Chan and Neece, 2018; Neece, 2014) positive results were found for only two programs (Triple P/SSTP, and MAPS).

### Limitations and future research

As research in general, our study has some limitations. The search terms narrowed the selection of studies for this review. Only studies published in English in peer-reviewed journals between January 1, 2010 and March 1, 2023 were included. The results of the studies that we reviewed were heterogeneous, with effect sizes ranging from no effect over the control group to very large effect sizes. Variation in results can arise from differences in the study characteristics, such as the population, intervention, or outcome measures. It can also arise from methodological differences such as bias or study design (NHMRC, 2019).

Heterogeneity of the disability status may have increased variation in the results. There were some concerns about the risk of bias in the studies. Indirectness of the evidence was increased by short-term evaluations and short follow-up periods. The small number of participants in the studies increased the imprecision of the evidence.

This review was limited because of its focus on the quantitative results of effectiveness of the included interventions. Qualitative analysis might shed more light on the experiences of the participants and benefits for the families.

Non-replicated studies of interventions should be replicated by further research to give recommendations for effective practices. Longer follow-up periods are warranted in future studies. Additional research is needed to understand the perspective of the child and the effects of parental interventions on the well-being of children with intellectual disabilities.

### Conclusions

To our knowledge, this is the first systematic review of psychological interventions for parents of children with intellectual disabilities which includes an intervention content analysis and an effect size analysis on parental well-being and child behavior. All the reviewed studies reported improvements in parental well-being or child behavior. However, effect sizes were small for the studies with the largest sample sizes. The most effective interventions were provided in a group format by professionals. The group format offers peer support, and parents receive feedback from other participants as well as from professional group leaders. At follow-up, most improvements in parental well-being and child behavior were sustained or further increased.

We categorized the interventions by their content into those targeting ‘Child or interaction’ (child behavior, interaction and learning, understanding disability), and those targeting ‘Parent’ (parental well-being) or both of these themes. Promising improvements were shown in interventions addressing the ‘Child or interaction’ based on theories and framings such as applied behavior analysis, cognitive behavior therapy, psychoeducation, social learning theory, and solution-focused systemic therapy; and in interventions focusing on the ‘Parent’ founded on mindfulness-based stress reduction, psychological well-being model, self-compassion and hope theories, and stress appraisal and coping model.

In the future, it may be beneficial to develop interventions which combine content focused on the child or interaction, with more intense focus on parental well-being based on the aforementioned theories. Combined interventions may enhance both parental well-being and child behavior, thus improving the well-being of the whole family. In addition, mentalization-based interventions should be developed and studied. It may also be worth developing and studying parenting interventions with parallel behavioral or well-being programs for children with intellectual disabilities.

Any positive effects on parental well-being and child behavior are needed in the vulnerable families of children with intellectual disabilities. Thus, it is crucial to ensure that parents of children with intellectual disabilities can reach and attend parental programs designed to improve the well-being of their families.

## Supplemental Material

Supplemental Material - Psychological interventions for parents of children with intellectual disabilities to enhance child behavioral outcomes or parental well-being: A systematic review, content analysis and effects Supplemental Material for Psychological interventions for parents of children with intellectual disabilities to enhance child behavioral outcomes or parental well-being: A systematic review, content analysis and effects by Kati Ranta, Heini Saarimäki, Johanna Gummerus, Jael Virtanen, Satu Peltomäki, Elina Kontu in Journal of Intellectual Disabilities

Supplemental Material - Psychological interventions for parents of children with intellectual disabilities to enhance child behavioral outcomes or parental well-being: A systematic review, content analysis and effects Supplemental Material for Psychological interventions for parents of children with intellectual disabilities to enhance child behavioral outcomes or parental well-being: A systematic review, content analysis and effects by Kati Ranta, Heini Saarimäki, Johanna Gummerus, Jael Virtanen, Satu Peltomäki, Elina Kontu in Journal of Intellectual Disabilities

Supplemental Material - Psychological interventions for parents of children with intellectual disabilities to enhance child behavioral outcomes or parental well-being: A systematic review, content analysis and effects Supplemental Material for Psychological interventions for parents of children with intellectual disabilities to enhance child behavioral outcomes or parental well-being: A systematic review, content analysis and effects by Kati Ranta, Heini Saarimäki, Johanna Gummerus, Jael Virtanen, Satu Peltomäki, Elina Kontu in Journal of Intellectual Disabilities

Supplemental Material - Psychological interventions for parents of children with intellectual disabilities to enhance child behavioral outcomes or parental well-being: A systematic review, content analysis and effects Supplemental Material for Psychological interventions for parents of children with intellectual disabilities to enhance child behavioral outcomes or parental well-being: A systematic review, content analysis and effects by Kati Ranta, Heini Saarimäki, Johanna Gummerus, Jael Virtanen, Satu Peltomäki, Elina Kontu in Journal of Intellectual Disabilities
