# Supplementary material for: Psychological interventions for parents of children with intellectual disabilities to enhance child behavioral outcomes or parental well-being: A systematic review, content analysis and effects
Source: J Intellect Disabil. 2024 Nov 27;29(2):500–35. doi: 10.1177/17446295241302857 (PMC12084672; doi:10.1177/17446295241302857)
Supplement: Supplemental Material - Psychological interventions for parents of children with intellectual disabilities to enhance child behavioral outcomes or parental well-being: A systematic review, content analysis and effects [file sj-pdf-1-jld-10.1177_17446295241302857.pdf]

Supplementary Figure 1. Risk of Bias Figure

Risk of bias in randomized controlled trials

Authors (Year)	Random sequence generation	Allocation concealment	Blinding of participants and personnel	Blinding of outcome assessment	Attrition	Selective reporting
**Randomized controlled trials**						
Chan and Neece (2018)	+	-	-	-	-	-
Coulman et al. (2022)	-	+	-	-	-	+
Grenier-Martin et al. (2022)	+	-	-	-	-	-
Hall et al. (2020)	-	-	-	-	-	-
Hamdani et al. (2021)	-	+	-	-	+	+
Hand et al. (2012)	-	+	-	-	+	+
Hinton et al. (2017)	+	-	-	-	+	+
Kleefman et al. (2014)	+	-	-	-	X	+
Kostulski et al. (2021)	+	-	-	-	+	+
Lee et al. (2022)	+	-	-	-	-	-
López-Liria et al. (2020)	-	-	-	-	-	+
Platje et al. (2018)	-	-	-	-	+	+
Roux et al. (2013)	+	+	-	-	+	+
Shapiro et al. (2014)	-	-	-	-	X	+
Sofronoff et al. (2011)	-	-	-	-	-	-
Yıldırım et al. (2012)	+	+	-	-	+	+
